# Mechanical Characteristics of Graphene Nanoplatelets-Modified Asphalt Mixes: A Comparison with Polymer- and Not-Modified Asphalt Mixes

**DOI:** 10.3390/ma14092434

**Published:** 2021-05-07

**Authors:** Laura Moretti, Nico Fabrizi, Nicola Fiore, Antonio D’Andrea

**Affiliations:** Department of Civil, Constructional and Environmental Engineering, Sapienza University of Rome, Via Eudossiana 18, 00184 Rome, Italy; fabrizi.1770990@studenti.uniroma1.it (N.F.); nicola.fiore@uniroma1.it (N.F.); antonio.dandrea@uniroma1.it (A.D.)

**Keywords:** modified asphalt, fatigue resistance, stiffness modulus, graphene nanoplatelets, nanomodification

## Abstract

In recent years, nanotechnology has sparked an interest in nanomodification of bituminous materials to increase the viscosity of asphalt binders and improves the rutting and fatigue resistance of asphalt mixtures. This paper presents the experimental results of laboratory tests on bituminous mixtures laid on a 1052 m-long test section built in Rome, Italy. Four asphalt mixtures for wearing and binder layer were considered: two polymer modified asphalt concretes (the former modified with the additive Superplast and the latter modified with styrene–butadiene–styrene), a “hard” graphene nanoplatelets (GNPs) modified asphalt concrete and a not-modified mixture. The indirect tensile strength, water sensitivity, stiffness modulus, and fatigue resistance of the mixtures were tested and compared. A statistical analysis based on the results has shown that the mixtures with GNPs have higher mechanical performances than the others: GNP could significantly improve the tested mechanical performances; further studies will be carried out to investigate its effect on rutting and skid resistance.

## 1. Introduction

Road pavement distresses depend on fatigue cracking and rutting damages: in flexible pavements both affect the bonded layers [1]. Their effects lead to a decrease in safety, durability, and efficiency of the infrastructure that have an impact on users and road agencies in terms of economic costs. Moreover, road infrastructure entails environmental impacts, in terms of abiotic resource depletion and emissions caused by roadworks [2,3,4]. For these reasons, in recent decades research has focused on chemical products able to increase durability and improve mechanical properties of pavements. The most frequently studied products are fibers [5,6,7], rubber [8,9,10], and a wide variety of additives (e.g., polymer, iron powder, hydrated lime, glass wastages [11,12,13,14,15]) and, more recently, nanomaterials [16,17]. Nanotechnology deals with the sub-nanometer dimensional scale; nanoparticle has its size between 1 to 100 nm. Physics and chemistry of nanometric particles differ from those of conventional materials due to the drastic increase in the surface area to volume ratio [18]. They are composed of high-performance materials that modify the molecular structure and improve mechanical properties and durability of materials [19]. In regard to flexible pavements, the mechanical behavior of bituminous materials depends on structural elements and phenomena occurring at the micro and nanometric scale [20]. Indeed, nanomaterials can improve the performance of binders and bound materials by providing better resistance to traffic and environmental loads, and they can also mitigate the incompatibility between some natural aggregates and bitumen, allowing more sustainable and long-lasting pavements [21,22,23]. Among the nano additives considered in the literature, the most promising products for bituminous mixtures are; anoclays (NC), nanosilicates, carbon nanotubes (CNTs), and graphene nanoplatelets (GNPs). Nanoclays are layered silicate nanoparticles, compatible with organic monomers and polymers, with a layer thickness of the order of 1 nm [24]; they are relatively inexpensive compared to polymeric additives [25] and have remarkable mechanical properties that increase the bitumen stiffness [26]. Nanosilicates are advantageous due to their low production cost and high performance; hey could improve resistance to aging, breaking, and cracking, but their low-temperature performances are not remarkable [24]. Carbon nanotubes (CNTs) are nanometer-scale graphene sheets rolled into hollow cylinders; they have a high mechanical performance [27] and their addition increases adhesive forces and reduces susceptibility to moisture [28]. Furthermore, CNTs can increase the resistance to fatigue and rutting, compared to conventional mixtures [29]. Meanwhile, the relatively high cost of CNTs limits their applications in flexible pavements. GNPs consist of stacked graphene layers that can be characterized as nanodiscs with a sub-micrometer diameter and thickness of about 1 nm. GNPs have remarkable mechanical properties [30,31,32] and low cost; moreover, their homogeneous dispersion within the bituminous matrix is simple [33]. The nanomodification of asphalt involves the binder used to produce the mixtures; nanomaterials are mixed with bitumen [34] and in some cases a modification with polymer is also performed, thus obtaining a polymer-modified nano binder (PMB) [35].

In the literature, several studies demonstrated that carbon nanoparticles and CNTs improve aging resistance and fatigue and rutting performances of bituminous binders [36,37]; the results show an improvement in the properties of the binders’ mixtures at high and low temperatures [38]. The modification mechanisms strongly depends on the type of additive, its dosage, and the adopted mixing procedure [39]. Having regard to asphalt mixtures, several studies have found that the modification of conglomerates with nanoclays, nanosilicates, and carbon nanotubes increases durability in the storage phase and resistance to aging, reduces water sensitivity, and improves mechanical properties at low temperatures and pavement durability [24]. The comparison between the mechanical properties of a not-modified and a modified mix reveals an increase in Marshall stability, higher indirect tensile strength, higher stiffness, lower permanent deformation, and better fatigue strength [40,41,42,43]. Moreover, nanomaterials can improve the aging resistance of binders because they can act as a barrier thus delaying the oxidation process and they can prevent the evaporation of the light components of bitumen [44].

This paper presents the first phase of a study carried out to comparative analysis of volumetric, physical, and mechanical characteristics and functional performances of not-modified and modified asphalt mixes used to build wearing and binder layers of a full-scale flexible pavement. The test section was built in the Metropolitan City of Rome: a 1 km-long stretch of the Provincial Road SP 03 Ardeatina has been laid. It was divided into four sections, whose wearing and binder layers were made with four asphalt mixes:

(1) Not-modified mixture;
(2) “Soft” modified asphalt concrete contains standard thermoplastic polymer additive Superplast (in this paper the terms “soft and “hard” refer to the lowest and a highest polymer additive content, respectively);
(3) “Hard” modified asphalt concrete contains SBS (styrene–butadiene–styrene);
(4) “Hard” GNP-modified asphalt concrete (this mixture contains the compound of polymers, recycled hard plastic, and GNPs Gipave^®^ by Iterchimica srl).

Each mix had Reclaimed Asphalt Pavement (RAP): 40% of removed binder and 30% of removed wearing layer were recycled. As a whole, the mixes differ only in the additive component; he aggregates come from the same quarry and have the same grading curve. In the first phase, the mixtures were subjected to characterization of particle size and study of volumetric properties. In the second phase, the physical–mechanical characteristics of the mixtures were evaluated, that is the indirect tensile strength and the water sensitivity. In the third phase, mechanical performances in terms of stiffness modulus and fatigue resistance of the mixtures were investigated. The study has been strengthened by statistical analyses based on the collected data and involving the calculation of the arithmetic mean, the standard deviation, and the coefficient of variation.

## 2. Materials and Methods

The analyzed bituminous mixtures were taken during the pavement construction phase of a 1052 m-long test section built on the Provincial Road SP 03 Ardeatina, between km 15 + 800 and km 16 + 852 into a southerly direction. The work consisted in the replacement of 3 cm thick wearing layer and 7 cm thick binder layer. Four different bituminous materials were considered, and the test section was divided into four sections for comparative purposes:

- Section 1 (S1) is 265 m long, between km 15 + 800 and km 16 + 065, is composed of modified asphalt concrete with GNPs;
- Section 2 (S2) is 179 m long, between km 16 + 121 and km 16 + 330, is composed of “hard” modified asphalt concrete with SBS;
- Section 3 (S3) is 228 m long, between km 16 + 332 and km 16 + 560, is composed of “soft” modified asphalt concrete with Superplast;
- Section 4 (S4) is 172 m long, between km 16 + 680 and km 16 + 825, is composed of not-modified asphalt concrete.

**Figure 1:**
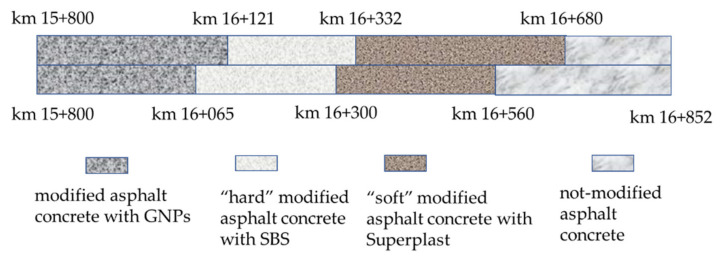
Longitudinal section of the experimental stretch.

Figure 1 shows the longitudinal section of the experimental stretch.

The composition of the tested mixes Mi is listed in Table 1.

The binder extraction according to the European standard EN 12697-1 allowed for determination of the soluble binder content in the mixtures [45]. Table 2 lists the results for the wearing and binder layers.

**Figure 2:**
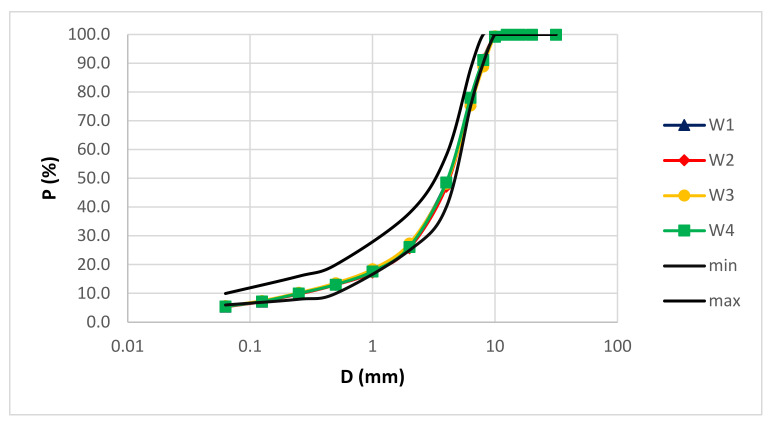
Aggregate grading of the wearing layers.

**Figure 3:**
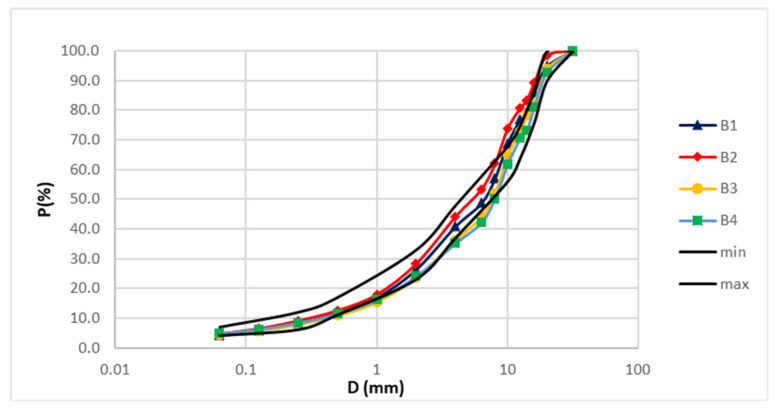
Aggregate grading of the binder layers.

Figure 2 and Figure 3 show the mixture aggregates grading of the wearing and binder layers, respectively.

In the wearing layer (Figure 2) the aggregate mixtures are continuously and homogeneously graded, while in the binder layer (Figure 3) a difference is appreciated due to coarse calcareous aggregates which in the compaction phase may have a slight crushing.

### 2.1. Volumetric Characteristics

The theoretical maximum density of the bituminous mixtures (*ρ_mc_*) has been calculated according to EN 12697-5 (Equation (1)):

(1) $$\rho_{mc}=\frac{100}{\left(\frac{p_{a}}{\rho_{a}}\right)+\left(\frac{p_{b}}{\rho_{b}}\right)}$$

where *p_a_* is the percentage of aggregate in the mixture, *ρ_a_* is the apparent density of the aggregate, *p_b_* is the percentage of binder in the mixture, and *ρ_b_* is the density of the binder at 25 °C, where (Equation (2))

*p_a_* + *p_b_* = 100% (2)

Cylindrical samples were prepared according to the European standard EN 12697-31, the bulk density (*ρ_bssd_*) has been evaluated according to the saturated surface dry method EN 12697-6, and the air voids content according to EN 12697-8.

### 2.2. Physical-Mechanical Properties

#### 2.2.1. Indirect Tensile Strength and Water Sensitivity

According to the European standard EN 12697-23, the indirect tensile strength (ITS) of the mixtures has been evaluated.

In order to investigate the durability and moisture susceptibility of the mixtures, the indirect tensile test has been carried out on dry and water conditioned specimens; the Indirect Tensile Strength Ratio (ITSR) is given by Equation (3) (EN 12697-12):

(3) $$\mathrm{ITSR}=\frac{ITS_{W}}{ITS_{d}}\cdot 100$$

where *ITS_W_* is the indirect tensile strength of specimens saturated and stored in water at 40 °C for 72 h, and *ITS_d_* is the indirect tensile strength of specimens stored dry at room temperature.

#### 2.2.2. Stiffness Modulus

The stress–strain response of pavement under traffic loading has been investigated according to the annex C of the standard EN 12697-26. The test was carried out on cylindrical specimens, adopting an indirect tensile configuration, and applying dynamic pulses with a haver-sinusoidal load shape and a fixed pulse repetition time of 3 s. The tests were performed in deformation control, calibrating pulses to generate a horizontal deformation of 5 μm in the specimens. To evaluate the sensitivity of the stiffness modulus (*S_m_*) to temperature, the tests were carried out at 5 °C, 25 °C, and 40 °C. Specimens with the same void content were tested in order to compare *S_m_* of asphalt mixtures that differ only for their binder (Equation (4)):

(4) $$S_{m}=\frac{F}{z\cdot h}\cdot \left(\nu +0.27\right)$$

where *F* is the maximum value of the applied load, *z* is the amplitude of the horizontal deformation, *h* is the average thickness of the specimen, and *ν* is the Poisson’s ratio.

#### 2.2.3. Fatigue Resistance

The fatigue strength has been performed according to the EN 12697-24 standard (Annex E), which allows determining the fatigue strength of cylindrical specimens of bituminous conglomerate in indirect tensile configuration. Dynamic pulses were applied with a haver-sinusoidal load shape, a load frequency of 2 Hz, and a test temperature of 25 °C were chosen. Three tests were carried out for each type of analyzed mixture, at different initial strain levels (ε_0_) between 100 με and 400 με. Once the specified initial horizontal diametral deformation has been reached, the test was stress controlled and the deformation trend was monitored from the fixed ε_0_. Two failure criteria were adopted to calculate the fatigue strength: the number of cycles in which the initial deformation doubled, and the number of load cycles in which the fracture of the specimen occurred, hence a sudden increase in deformation. For each type of bituminous mixture, the trend of the stiffness modulus was evaluated as the load cycles vary and finally the fatigue curves were represented.

## 3. Results

### 3.1. Volumetric Characteristics

Ten specimens were molded for each mixture using gyratory compactor in order to evaluate voids contents. The results in Table 3 showed air voids in a relatively narrow range (i.e., minimum 5.65% and maximum 6.61%) for wearing mixes. A wider range (i.e., minimum 4.85% and maximum 6.99%) was obtained for binder mixes, although it should be noted that the modified mixes (i.e., B1 to B3) showed very close air voids, whereas the not-modified binder mix (i.e., B4) voids content was quite higher than modified ones probably due to the lower content of bitumen. All mixes showed low variability in terms of air voids, as confirmed by the standard deviations in Table 3.

**Figure 4:**
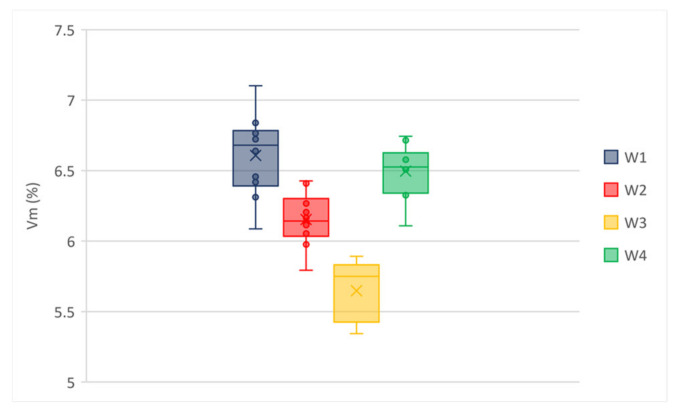
Voids content of wearing mixtures.

**Figure 5:**
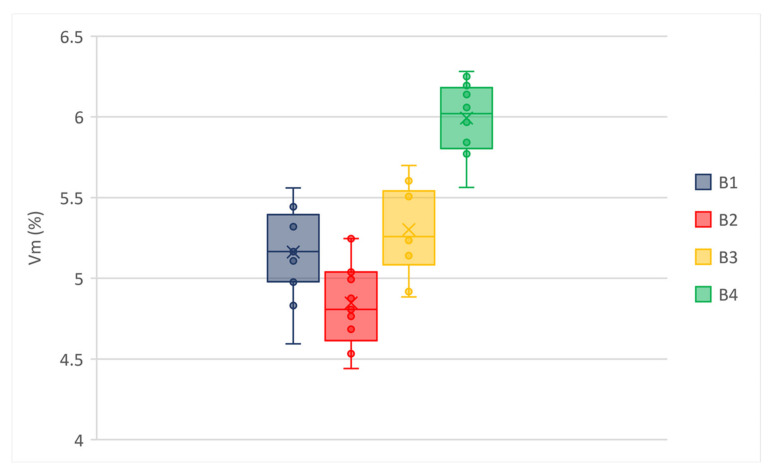
Voids content of binder mixtures.

The box and whisker plots in Figure 4 and Figure 5 permit the explanatory data analysis of the average voids content of the wearing and binder mixtures, respectively. The distribution of numerical data demonstrates that the experimental data set is normally distributed, without observations that are numerically distant from the rest of the data (i.e., outliers). Most of median line of each box plot lies outside of the other boxes; it confirms that there is likely a difference between the tested materials.

### 3.2. Physical and Mechanical Characteristics

#### 3.2.1. Indirect Tensile Strength

Table 4 lists the average ITS values obtained from six parallel Marshall specimens prepared in the laboratory for each mix.

**Figure 6:**
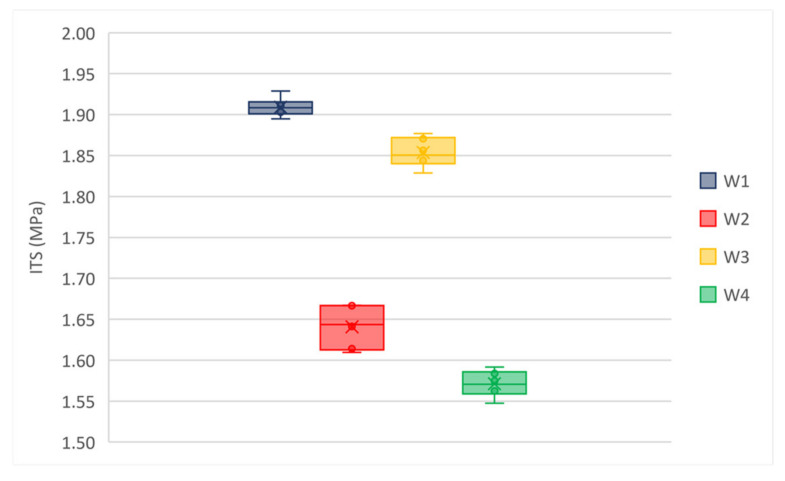
Average ITS of wearing mixtures, T = 25 °C.

**Figure 7:**
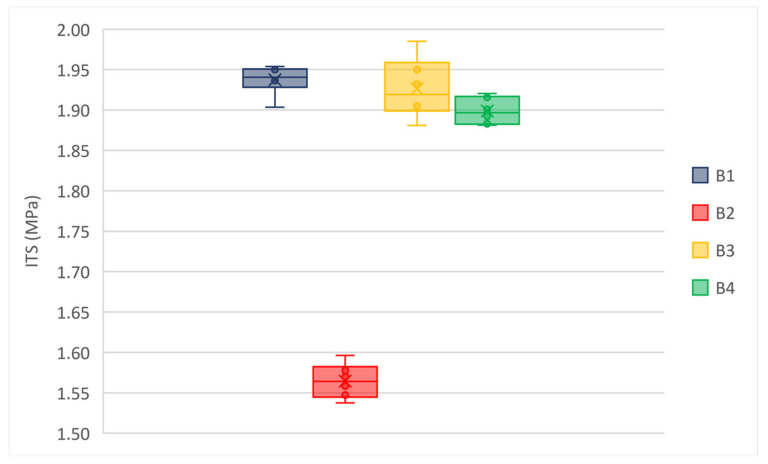
Average ITS of binder mixtures, T = 25 °C.

Figure 6 and Figure 7 represent the ITS values for wearing and binder mixtures, respectively.

The box and whisker plots in Figure 6 and Figure 7 highlight that there are not outliers in the experimental data set. Both for the wearing and binder mixtures the median line of each box plot lies outside of the other boxes. ITS values at 25 °C is sensitive to mixture properties (e.g., air voids, asphalt binder content, binder grade, and aggregates); it justifies the observed differences. However, all mixtures showed high values of ITS that could result in low cracking resistance and fragile behavior of materials. Therefore, this data set has been compared with results from stiffness modulus and the fatigue resistance tests.

#### 3.2.2. Water Sensitivity

For each Mi mix, a set of six Marshall specimens was selected to evaluate the indirect tensile strength ratio as a performance indicator for water sensitivity of asphalt. All the specimens intended for these tests were compacted using a Marshall hammer with 35 blows per face. Table 5 lists the obtained ITSR indices, their average, and standard deviation values.

**Figure 8:**
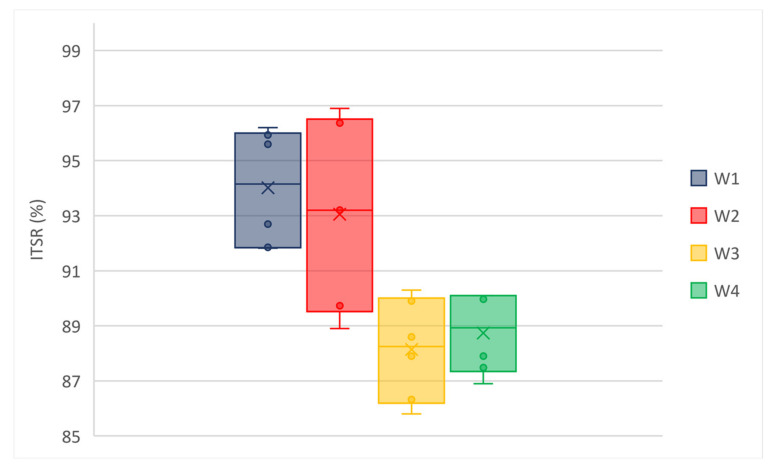
ITSR of wearing mixtures.

**Figure 9:**
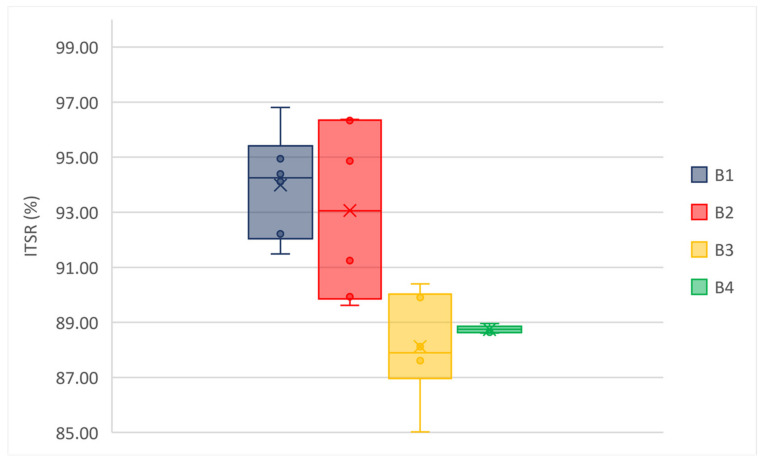
ITSR of binder mixtures.

Low values of standard deviation indicate that the values tend to be close to the average; the coefficient of variation (i.e., the ratio between the standard deviation and the average) is much less than 1 for each Mi. Figure 8 and Figure 9 show the box and whisker plots of ITSR values for wearing and binder mixtures, respectively.

All the mixtures showed a reduced sensitivity to water since they had no significant drops in ITS after conditioning. The observed trend of ITSR demonstrates that the water sensitivity is increasing from M1 to M3, while the performances of M3 and M4 are comparable. Moreover, an outlier value is observed for B4 (i.e., 92.57% for specimen 2#). The results indicate a lower sensitivity to water, a lower loss of performance, and a greater durability of the mixtures modified with GNPs.

#### 3.2.3. Stiffness Modulus

The stiffness modulus tests were carried out at different temperatures (i.e., 5 °C, 25 °C, and 40 °C). For each temperature and mixture, four specimens were tested, and the average results of stiffness modulus are listed in Table 6. Specimens with the same voids content were selected to carry out the tests, because both the stiffness modulus and the fatigue resistance are seriously affected by V_m_. The statistical analysis of the data set revealed that the test results were reliable because testing variation for each mixture was small (i.e., the standard deviation was low and the coefficient of variation was much less than 1 for each Mi).

**Figure 10:**
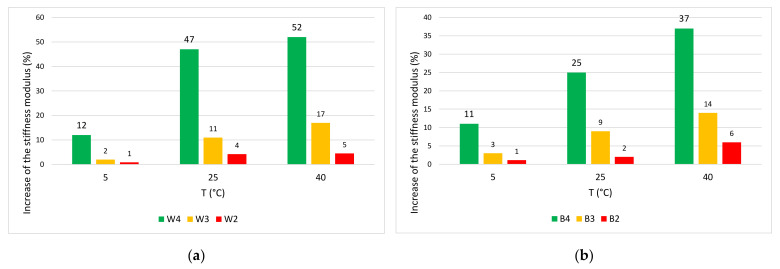
Increase of the stiffness modulus compared to M1 (**a**) wearing and (**b**) binder.

The mixtures prepared with modified bitumen have higher stiffness modulus values than those prepared with traditional bitumen. It was also noted that the mixtures modified with GNPs showed, at all tested temperatures, values of the stiffness modulus greater than the others. The higher stiffness is due to the elastic properties of GNP modified mixtures. The increase of the stiffness modulus compared to M1 is in Figure 10a,b for wearing and binder layers, respectively.

In the GNP-modified wearing, at 40 °C the increase is +52% compared to W4, +17% compared to the soft mixture (W3), and +5% compared to the hard mixture (W2). The increase in the modulus achieved in M1 is remarkable compared to M4. A slight increase is observed compared to M3 and M2. This result is most noticeable at high temperatures. The increase in stiffness modulus at high temperatures reduces tensile deformations induced by traffic loads. The higher elasticity and the greater resistance of the mixture is due to the large specific surface and mechanical properties of GNPs [31,32,37,46].

#### 3.2.4. Fatigue Resistance

**Figure 11:**
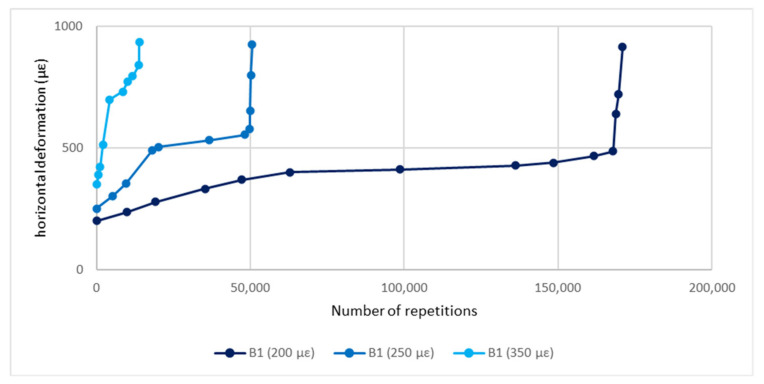
Deformation curves of B1 for different initial strain values.

Finally, fatigue tests with different initial strain values (i.e., 200 με, 250 με, and 350 με) have been carried out on four specimens of each mixture conditioned at 25 °C. Figure 11 shows the average relationships between the number of load applications and the total horizontal deformation in B1.

**Figure 12:**
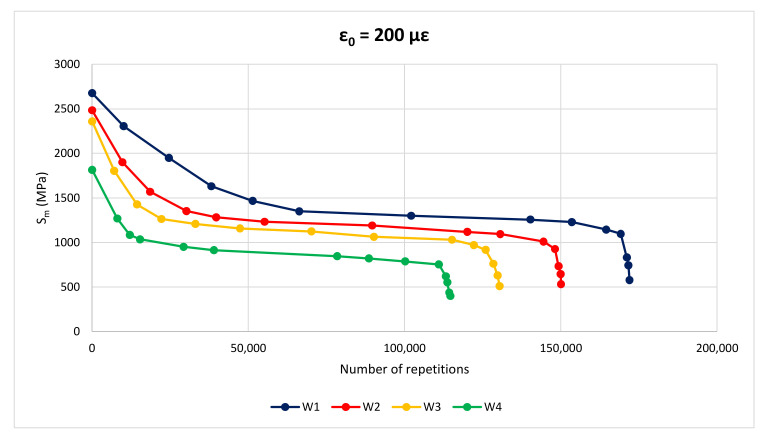
Fatigue curve of wearing layers, ε_0_ = 200 με.

**Figure 13:**
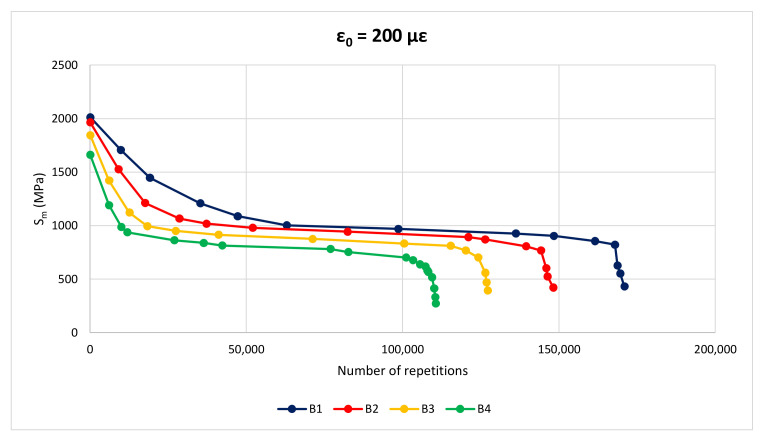
Fatigue curve of binder layers, ε_0_ = 200 με.

In Figure 11 there are three distinct regions: in the initial part the slope of the deformation curve decreases with increasing number of loading repetitions, in the middle part the slope of the curve is quite flat, and in the last part the slope increases with increasing number of loading cycles causing a fast and complete fracture of the specimen. The first two phases refer to the crack initiation, while the third one describes the crack propagation, when transition of microcracks into macrocracks occurs. For all the mixtures, the evolution of the modulus in the first phase is greatly affected by the initial strain (ε_0_); the greater ε_0_ the greater the decrease of the complex modulus. Figure 12 and Figure 13 show stiffness curves for ε_0_ = 200 με for wearing and binder layers, respectively.

At any ε_0_, the initial part of the curves highlights a minor decrease in stiffness modulus for M1 mixtures compared to the others. Moreover, the middle part of GNP-modified mixtures is longer in terms of number of load repetitions before failure.

Table 7 and Table 8 list all the average fatigue test results for wearing and binder layers, respectively, where N(2ε_0_) is the number of repetitions to double the initial strain and N_f_ is the number of repetitions to have a failure.

**Figure 14:**
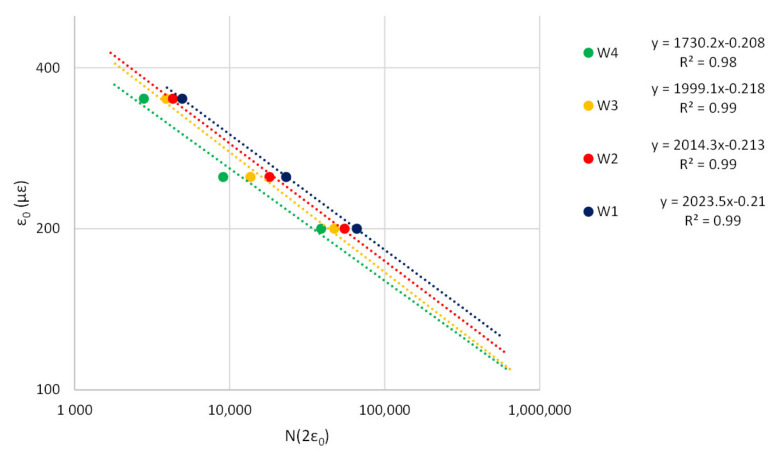
Fatigue curves—wearing mixtures.

**Figure 15:**
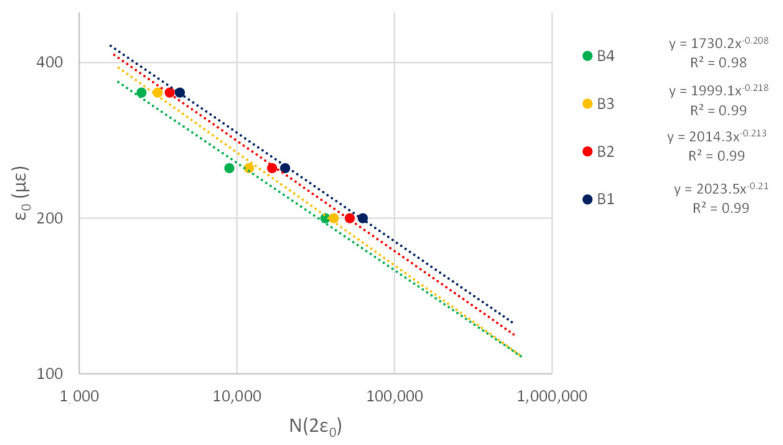
Fatigue curves—binder mixtures.

In this study, strain increase by a factor of two over its initial value has been adopted as failure criterion. Data from Table 7 and Table 8 allowed definition of the fatigue curves for wearing and binder layers in Figure 14 and Figure 15, respectively.

The fatigue curves highlight M1 mixtures (i.e., the blue curves in Figure 14 and Figure 15) have the best performances, while M4 ensure the lowest resistance to fatigue (i.e., the green curves in Figure 14 and Figure 15). M1 mixtures offer slightly better fatigue strength than modified mixtures with SBS (i.e., M2) and Superplast (i.e., M3). Table 9 and Table 10 detail the percentage increase of load repetitions compared to W1 and B1 mixtures calculated as the ratio between the number of load repetitions to double ε_0_ in the examined mixture (N_Wx,2ε0_ for the wearing layer) or to achieve failure (N_Wx,f_ for the wearing layer) and the reference (i.e., that with GNPs) (N_W1,2ε0_ or N_W1,f_, respectively, for the wearing layer).

The increase in fatigue resistance is due to the less propagation of microcracks and to the self-healing properties provided by the GNP modifier compound.

## 4. Conclusions

In the past decade, nanotechnology has been explored in a wide range of disciplines with the “bottom-up” engineering approach; nanomodification technology aims to influence the mass properties of materials leading to new applications or enhanced utilities using chemical or physical properties operating at the nanoscale.

This article studies the mechanical properties of a “hard” graphene nanoplatelets (GNPs) modified asphalt mixture compared to a not- modified and two polymer-modified asphalt mixtures (the former mix is a “soft” modified asphalt concrete with Superplast; the latter is a “hard” modified asphalt concrete with SBS). Eight mixtures—four wearing and four for binder layers—have been analyzed. All the mixtures were sampled during the construction of a 1052 m-long test stretch composed of four sections for comparative purposes. Particularly, the mixtures were laboratory evaluated in terms of volumetric, physical, and mechanical performances; the void contents, indirect tensile strength, water sensitivity, stiffness modulus, and fatigue resistance were tested on several specimens of each mixture. Based on the analysis of the results obtained from the laboratory tests, all the mixtures show high values of indirect tensile strength and low water sensitivity; however, the GNP modified mix shows a higher ITRS and a lower drop in ITS after conditioning. GNP modified mixes also show higher stiffness especially at higher temperatures when compared with the not-modified asphalt mix; this means they are suitable for warm climates. Fatigue resistance has been monitored as loss in stiffness modulus vs. number of repetitions; GNP modified mixes show a slower stiffness decrease at any initial strain level according to EN 12697-24 (Annex E). The fatigue curves highlight that GNP mixtures have better performances than the not-modified asphalt concrete and slightly better even than the mixtures modified with SBS and Superplast.

The results showed that GNPs improve the mechanical performance of the modified asphalt mixture and its durability. The high surface area of GNPs increases the pavement’s bonding strength and makes the asphalt binder stiffer. Therefore, the binder modification at the nanoscale can optimize the performance of bituminous materials, with a consequent improvement in mechanical properties and durability of road pavements.

The current research on the use of nanomaterials in asphalt mixtures is composed of two phases: the former involves the laboratory characterization of laid materials; the latter will include the investigation of the built pavements exposed to traffic. This paper presents the findings of the first phase only. The investigation in the second phase will involve the comparison of structural and functional properties of the considered wearing layers having regard to their skid and rutting resistance, respectively. Moreover, further research could be developed to compare high-performance mixtures and assess the environmental impacts and maintenance costs of road pavements managed with nanotechnologies.

## Figures and Tables

**Table 1:** Composition of the tested mixes.

Mix ID	ID Layer	Layer	Type	ID Modifier	Modifier/Bitumen (% by Weight)	RAP Surface Layer (%)	Renovator/RAP (%)
M1	W1	wearing	GNP based compound	GNP	5	30	0.2
	B1	binder				40	
M2	W2	wearing	hard	SBS	5	30	
	B2	binder				40	
M3	W3	wearing	soft	Superplast	3	30	
	B3	binder				40	
M4	W4	wearing	not modified	-	-	30	
	B4	binder				40	

**Table 2:** Bitumen content.

Layer	ID Mix	Bitumen/Mixture (%)
wearing	W1	5.95
	W2	6.45
	W3	6.62
	W4	6.32
binder	B1	4.78
	B2	5.03
	B3	4.41
	B4	3.80

**Table 3:** Air voids of the mixtures.

Layer	ID Mix	Average Voids (%)	Standard Deviation (%)
wearing	W1	6.61	0.293
	W2	6.15	0.192
	W3	5.65	0.207
	W4	6.50	0.192
binder	B1	5.16	0.272
	B2	4.85	0.259
	B3	5.30	0.278
	B4	5.99	0.218

**Table 4:** ITS values of the mixtures.

Layer	ID mix	1#	2#	3#	4#	5#	6#	Average (MPa)	Standard Deviation (MPa)
wearing	W1	1.91	1.93	1.91	1.90	1.89	1.91	1.92	0.011
	W2	1.67	1.61	1.64	1.67	1.65	1.61	1.63	0.025
	W3	1.86	1.88	1.87	1.83	1.84	1.84	1.85	0.018
	W4	1.59	1.58	1.57	1.57	1.55	1.56	1.59	0.016
binder	B1	1.95	1.94	1.94	1.95	1.94	1.90	1.94	0.018
	B2	1.58	1.57	1.56	1.60	1.54	1.55	1.56	0.021
	B3	1.88	1.90	1.99	1.91	1.93	1.95	1.93	0.037
	B4	1.92	1.89	1.88	1.88	1.90	1.92	1.91	0.016

**Table 5:** ITSR values of the mixtures.

Layer	ID Mix	1#	2#	3#	4#	5#	6#	Average (%)	Standard Deviation (%)
wearing	W1	96.2	91.8	91.8	95.9	95.6	92.7	94.0	1.23
	W2	89.7	96.3	88.9	93.2	96.9	93.2	93.1	1.41
	W3	89.9	86.3	87.9	85.8	88.6	90.3	88.1	1.14
	W4	87.4	89.9	86.9	90.1	87.9	90.1	88.7	2.84
binder	B1	94.39	96.80	94.11	94.94	92.22	91.49	94.1	0.86
	B2	89.93	96.37	91.25	89.62	94.86	96.33	92.7	1.26
	B3	89.91	85.02	90.40	87.67	87.61	88.11	90.8	1.23
	B4	88.61	92.57	88.64	88.96	88.75	88.75	90.6	1.63

**Table 6:** Average values of stiffness modulus (S) at 5 °C, 25 °C, 40 °C and void contents.

ID mix	Stiffness Modulus (MPa)						V_m_ (%)
	T = 5 °C		T = 25 °C		T = 40 °C		
	Average	Standard Deviation (MPa)	Average	Standard Deviation (MPa)	Average	Standard Deviation (MPa)	
W4	7843	35	1807	7	895	49	5.6
W3	8559	21	2383	35	1170	19	5.7
W2	8682	36	2538	34	1302	20	5.5
W1	8760	76	2649	32	1364	32	5.6
B4	7738	20	1612	10	746	40	5.3
B3	8320	26	1840	12	898	4	5.3
B2	8499	30	1976	22	960	19	5.4
B1	8565	56	2010	33	1020	44	5.2

**Table 7:** Results of fatigue tests—wearing mixtures.

Layer	ε_0_ (µε)	σ_0_ (kPa)	N(2ε_0_)	N_f_
W4	200	185	39,010	114,720
W3		230	47,360	130,440
W2		370	55,230	150,100
W1		230	66,310	172,010
W4	250	280	9100	25,900
W3		390	13,630	34,220
W2		240	18,100	48,790
W1		300	23,180	56,130
W4	300	420	2800	10,250
W3		260	3910	13,990
W2		330	4310	15,740
W1		440	4950	17,330

**Table 8:** Results of fatigue tests—binder mixtures.

Layer	ε_0_ (µε)	σ_0_ (kPa)	N(2ε_0_)	N_f_
B4	200	185	36,410	110,640
B3		200	41,150	127,250
B2		320	52,100	148,210
B1		180	62,980	170,940
B4	250	220	8950	23,290
B3		330	11,910	31,100
B2		180	16,720	44,640
B1		230	20,220	50,550
B4	300	335	2480	9950
B3		190	3150	11,070
B2		230	3750	12,650
B1		335	4350	14,050

**Table 9:** Percentage increase of load repetitions compared to W1.

Layer	ε_0_ (με)	N_Wx,2ε0_/N_W1,2ε0_	N_Wx,f_/N_W1,f_
W4	200	70%	50%
	250	155%	117%
	350	77%	69%
W3	200	40%	32%
	250	70%	64%
	350	27%	24%
W2	200	20%	15%
	250	28%	15%
	350	15%	10%

**Table 10:** Percentage increase of load repetitions compared to B1.

Layer	ε_0_ (με)	N_Bx,2ε0_/N_B1,2ε0_	N_Bx,f_/N_B1,f_
B4	200	73%	55%
	250	126%	117%
	350	75%	41%
B3	200	53%	34%
	250	70%	63%
	350	38%	27%
B2	200	21%	15%
	250	21%	13%
	350	16%	11%

## Data Availability

All data is contained within the article.

## References

[1] Tozzo C., D’Andrea A., Al-Qadi I.L. (2015). Prediction of fatigue failure at asphalt concrete layer interface from monotonic testing. Transp. Res. Rec..

[2] Moretti L., Mandrone V., D’Andrea A., Caro S. (2018). Evaluation of the environmental and human health impact of road construction activities. J. Clean. Prod..

[3] Trunzo G., Moretti L., D’Andrea A. (2019). Life cycle analysis of road construction and use. Sustainability.

[4] Moretti L., Di Mascio P., D’Andrea A. (2013). Environmental impact assessment of road asphalt pavements. Mod. Appl. Sci..

[5] Gupta A., Rodriguez-Hernandez J., Castro-Fresno D. (2019). Incorporation of additives and fibers in porous asphalt mixtures: A review. Materials.

[6] Slebi-Acevedo C.J., Lastra-González P., Pascual-Muñoz P., Castro-Fresno D. (2019). Mechanical performance of fibers in hot mix asphalt: A review. Constr. Build. Mater..

[7] Abiola O.S., Kupolati W.K., Sadiku E.R., Ndambuki J.M. (2014). Utilisation of natural fibre as modifier in bituminous mixes: A review. Constr. Build. Mater..

[8] Behnood A. (2020). A review of the warm mix asphalt (WMA) technologies: Effects on thermo-mechanical and rheological properties. J. Clean. Prod..

[9] Wang T., Xiao F., Amirkhanian S., Huang W., Zheng M. (2017). A review on low temperature performances of rubberized asphalt materials. Constr. Build. Mater..

[10] Fiore N., Caro S., D’Andrea A., Scarsella M. (2017). Evaluation of bitumen modification with crumb rubber obtained through a high pressure water jet (HPWJ) process. Constr. Build. Mater..

[11] Behnood A., Modiri Gharehveran M. (2019). Morphology, rheology, and physical properties of polymer-modified asphalt binders. Eur. Polym. J..

[12] Balaguera A., Carvajal G.I., Albertí J., Fullana-i-Palmer P. (2018). Life cycle assessment of road construction alternative materials: A literature review. Resour. Conserv. Recycl..

[13] Polacco G., Filippi S., Merusi F., Stastna G. (2015). A review of the fundamentals of polymer-modified asphalts: Asphalt/polymer interactions and principles of compatibility. Adv. Colloid Interface Sci..

[14] Kalantar Z.N., Karim M.R., Mahrez A. (2012). A review of using waste and virgin polymer in pavement. Constr. Build. Mater..

[15] Shafabakhsh G., Taghipoor M., Sadeghnejad M., Tahami S.A. (2015). Evaluating the effect of additives on improving asphalt mixtures fatigue behavior. Constr. Build. Mater..

[16] Fusco R., Moretti L., Fiore N., D’andrea A. (2020). Behavior evaluation of bituminous mixtures reinforced with nano-sized additives: A review. Sustainability.

[17] Crucho J., Picado-Santos L., Neves J., Capitão S. (2019). A review of nanomaterials’ effect on mechanical performance and aging of asphalt mixtures. Appl. Sci..

[18] Teizer J., Venugopal M., Teizer W., Felkl J. (2012). Nanotechnology and Its Impact on Construction: Bridging the Gap between Researchers and Industry Professionals. J. Constr. Eng. Manag..

[19] Santagata E., Baglieri O., Tsantilis L., Chiappinelli G., Dalmazzo D. (2016). Bituminous-based nanocomposites with improved high-temperature properties. Compos. Part B Eng..

[20] Galooyak S.S., Dabir B., Nazarbeygi A.E., Moeini A. (2010). Rheological properties and storage stability of bitumen/SBS/montmorillonite composites. Constr. Build. Mater..

[21] Steyn W.J. (2011). Applications of Nanotechnology in Road Pavement Engineering. Nanotechnology in Civil Infrastructure.

[22] Santagata E., Baglieri O., Tsantilis L., Dalmazzo D. (2012). Rheological Characterization of Bituminous Binders Modified with Carbon Nanotubes. Procedia Soc. Behav. Sci..

[23] Moreno-Navarro F., Sol-Sánchez M., Gámiz F., Rubio-Gámez M.C. (2018). Mechanical and thermal properties of graphene-modified asphalt binders. Constr. Build. Mater..

[24] Yang J., Tighe S. (2013). A Review of Advances of Nanotechnology in Asphalt Mixtures. Procedia Soc. Behav. Sci..

[25] Yao H., Li L., Xie H., Dan H.-C., Yang X.-L. (2011). Microstructure and Performance Analysis of Nanomaterials Modified Asphalt. Proceedings of the Road Materials and New Innovations in Pavement Engineering.

[26] Hossain Z., Zaman M., Saha M.C., Hawa T. (2014). Evaluation of Viscosity and Rutting Properties of Nanoclay-Modified Asphalt Binders. Proceedings of the Geo-Congress 2014 Technical Papers.

[27] Introduction to Nanoscience and Nanotechnology—1st Edition—Gabor L. https://www.routledge.com/Introduction-to-Nanoscience-and-Nanotechnology/Hornyak-Tibbals-Dutta-Moore/p/book/9781420047790.

[28] Yusoff N.I.M., Breem A.A.S., Alattug H.N.M., Hamim A., Ahmad J. (2014). The effects of moisture susceptibility and ageing conditions on nano-silica/polymer-modified asphalt mixtures. Constr. Build. Mater..

[29] Arabani M., Faramarzi M. (2015). Characterization of CNTs-modified HMA’s mechanical properties. Constr. Build. Mater..

[30] Kim K., Regan W., Geng B., Alemán B., Kessler B.M., Wang F., Crommie M.F., Zettl A. (2010). High-temperature stability of suspended single-layer graphene. Phys. Status Solidi Rapid Res. Lett..

[31] Lee C., Wei X., Kysar J.W., Hone J. (2008). Measurement of the elastic properties and intrinsic strength of monolayer graphene. Science.

[32] Novoselov K.S., Geim A.K., Morozov S.V., Jiang D., Katsnelson M.I., Grigorieva I.V., Dubonos S.V., Firsov A.A. (2005). Two-dimensional gas of massless Dirac fermions in graphene. Nature.

[33] Brcic H. (2016). Investigation of the Rheological Properties of Asphalt Binder Containing Graphene Nanoplatelets. Master’s Thesis.

[34] Martinho F.C.G., Farinha J.P.S. (2019). An overview of the use of nanoclay modified bitumen in asphalt mixtures for enhanced flexible pavement performances. Road Mater. Pavement Des..

[35] You Z., Mills-Beale J., Foley J.M., Roy S., Odegard G.M., Dai Q., Goh S.W. (2011). Nanoclay-modified asphalt materials: Preparation and characterization. Constr. Build. Mater..

[36] Jamal Khattak M., Khattab A., Rizvi H.R. (2013). Characterization of carbon nano-fiber modified hot mix asphalt mixtures. Constr. Build. Mater..

[37] Nazari H., Naderi K., Moghadas Nejad F. (2018). Improving aging resistance and fatigue performance of asphalt binders using inorganic nanoparticles. Constr. Build. Mater..

[38] Santagata E., Baglieri O., Tsantilis L., Chiappinelli G. (2015). Fatigue and healing properties of nano-reinforced bituminous binders. Int. J. Fatigue.

[39] Liu K., Zhang K., Wu J., Muhunthan B., Shi X. (2018). Evaluation of mechanical performance and modification mechanism of asphalt modified with graphene oxide and warm mix additives. J. Clean. Prod..

[40] Cai L., Shi X., Xue J. (2018). Laboratory evaluation of composed modified asphalt binder and mixture containing nano-silica/rock asphalt/SBS. Constr. Build. Mater..

[41] Golestani B., Nam B.H., Moghadas Nejad F., Fallah S. (2015). Nanoclay application to asphalt concrete: Characterization of polymer and linear nanocomposite-modified asphalt binder and mixture. Constr. Build. Mater..

[42] Crucho J.M.L., das Neves J.M.C., Capitão S.D., de Picado-Santos L.G. (2018). Mechanical performance of asphalt concrete modified with nanoparticles: Nanosilica, zero-valent iron and nanoclay. Constr. Build. Mater..

[43] Sun L., Xin X., Ren J. (2017). Asphalt modification using nano-materials and polymers composite considering high and low temperature performance. Constr. Build. Mater..

[44] Fang C., Yu R., Liu S., Li Y. (2013). Nanomaterials applied in asphalt modification: A review. J. Mater. Sci. Technol..

[45] Ziyani L., Boulangé L., Nicolaï A., Mouillet V. (2017). Bitumen extraction and recovery in road industry: A global methodology in solvent substitution from a comprehensive review. J. Clean. Prod..

[46] Te Hsieh C., Chen J.M., Kuo R.R., Lin T.S., Wu C.F. (2005). Influence of surface roughness on water- and oil-repellent surfaces coated with nanoparticles. Appl. Surf. Sci..

